# DAMPening Inflammation by Modulating TLR Signalling

**DOI:** 10.1155/2010/672395

**Published:** 2010-07-13

**Authors:** A. M. Piccinini, K. S. Midwood

**Affiliations:** Kennedy Institute of Rheumatology Division, Faculty of Medicine, Imperial College of Science, Technology and Medicine, 65 Aspenlea Road, Hammersmith, London W6 8LH, UK

## Abstract

Damage-associated molecular patterns (DAMPs) include endogenous intracellular molecules released by activated or necrotic cells and extracellular matrix (ECM) molecules that are upregulated upon injury or degraded following tissue damage. DAMPs are vital danger signals that alert our immune system to tissue damage upon both infectious and sterile insult. DAMP activation of Toll-like receptors (TLRs) induces inflammatory gene expression to mediate tissue repair. However, DAMPs have also been implicated in diseases where excessive inflammation plays a key role in pathogenesis, including rheumatoid arthritis (RA), cancer, and atherosclerosis. TLR activation by DAMPs may initiate positive feedback loops where increasing tissue damage perpetuates pro-inflammatory responses leading to chronic inflammation. Here we explore the current knowledge about distinct signalling cascades resulting from self TLR activation. We also discuss the involvement of endogenous TLR activators in disease and highlight how specifically targeting DAMPs may yield therapies that do not globally suppress the immune system.

## 1. The Danger Hypothesis

Both infection and sterile tissue injury generate strong immune responses. This paradox was first resolved by Matzinger in 1994 who proposed that our immune system is designed to combat danger, rather than mediate recognition of non-self over self [1]. Pathogen-associated molecular patterns (PAMPs) and endogenous molecules created upon tissue injury, since called damage-associated molecular patterns (DAMPs), signal the threat of either infection or injury to the organism, independently of their non-self- or self-identity [2–5]. Among the cellular receptors that sense these danger signals, Toll-like receptors (TLRs) represent a key molecular link between tissue injury, infection, and inflammation. In the last decade a number of endogenous molecules specifically generated upon tissue injury that activate TLRs have been identified. Some are intracellular molecules normally inaccessible to the immune system that are released into the extracellular milieu as a result of cell necrosis or activation following injury. Others are extracellular matrix (ECM) molecule fragments that are released upon tissue damage or ECM molecules that are specifically upregulated in response to tissue injury [6]. 

In addition to playing a key role in host defence against danger, activation of TLRs has been linked to the pathogenesis of many inflammatory and autoimmune diseases including sepsis, rheumatoid arthritis (RA), systemic lupus erythematosus (SLE), inflammatory bowel disease (IBD), type I diabetes, and multiple sclerosis (MS). Hence, in recent years TLRs and associated signalling molecules have become attractive targets for their treatment and a number of inhibitors are currently in development or have progressed to clinical trials. Aberrant TLR activation is also thought to contribute to diseases with a strong association with inflammation such as cancer and atherosclerosis (reviewed in [7–11]). 

One of the key questions to emerge from these studies is what factors drive TLR activation during the progression of disease. There is an increasing body of evidence to suggest that DAMP-mediated inflammation plays a vital role. It is also becoming apparent that PAMPs and DAMPs act in quite a different manner in order to stimulate an immune response. Here we review the mechanisms of DAMP recognition by TLRs, the signalling cascades, and the biological outcomes resulting from self TLR activation, focusing on the differences to non-self TLR activation. We also discuss the evidence that implicates endogenous molecules in pathological TLR activation and examine how blockade of DAMP action may be therapeutically beneficial. Understanding more about the differences between PAMP- and DAMP-induced inflammation may enable us to specifically target inappropriate, pathogenic inflammation whilst leaving the host defence intact.

## 2. Endogenous Activators of TLRs

The first report of a putative endogenous activator of TLRs dates back to 2000, when heat shock protein 60 (HSP60) was shown to induce cytokine synthesis through TLR4 activation [12]. In the same year necrotic cells were found to induce pro-inflammatory and tissue repair gene synthesis and cause DC maturation in a TLR2 dependent manner, as a result of the release of their intracellular contents [13, 14]. The list of endogenous TLR2 and 4 activators has expanded quickly and encompasses other intracellular molecules such as heat shock proteins including HSP70, Gp96 [15–17], HSP22, and HSP72 [18, 19] and high-mobility group box-1 protein (HMGB1) [20–22] as well as ECM molecules such as biglycan [23], tenascin-C [24], versican [25], and fragments of ECM molecules including oligosaccharides of hyaluronic acid (HA) [26] and heparan sulfate (HS) [27]. 

**Figure 1:**
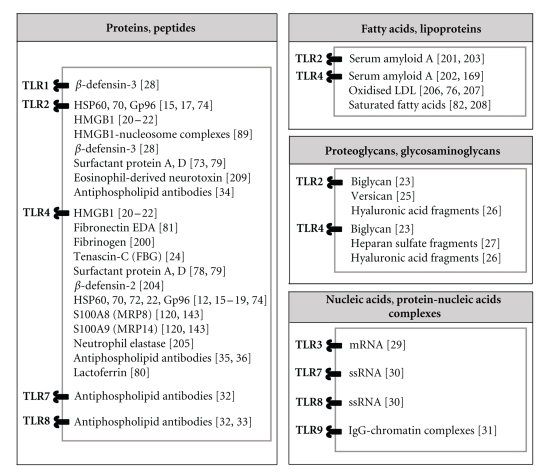
*Endogenous TLR activators*. TLRs are activated by damage-associated molecular patterns (DAMPs) including intracellular molecules released in the extracellular milieu by activated or necrotic cells and extracellular matrix molecules either upregulated upon injury or degraded following tissue damage. Known endogenous TLR activators are listed based on their biochemical nature.

Notably, the list of TLRs activated by endogenous molecules is also expanding. For instance, TLR1 was shown for the first time to be required, along with TLR2, for the activation of professional antigen-presenting cells by *β*-defensin-3, a host-derived antimicrobial peptide [28]. Self-nucleic acids have also been described as endogenous danger signals, namely, mRNA recognised by TLR3 [29], single-stranded RNA (ssRNA) sensed by TLR7 and 8 [30], and IgG-chromatin complexes recognised by TLR9 [31]. Interestingly, emerging data support the activation of TLR7 and 8 by antiphospholipid antibodies (APL) isolated from patients with APL syndrome [32, 33], as has been also shown previously for TLR2 and 4 [34–36]. A more complete list of DAMPs and their cognate TLRs can be found in Figure 1.

Given the use of *E. coli* to produce many of these endogenous molecules recombinantly, and the fact that most endogenous proteins activate TLR2 and 4, originally described as sensors of microbial products such as lipopolysaccharides (LPSs) and lipoproteins, the question of whether microbial contamination can partially or wholly account for DAMP activity remains a key issue. Erridge and Samani recently showed that apparent stimulation of TLR4 by saturated fatty acids was due to microbial contamination in their preparations of BSA [37]. In contrast, professional antigen-presenting cells that are not responsive to LPS were shown to be activated by necrotic cells indicating that LPS independent TLR4 activation does occur in response to endogenous ligands [38]. Similar to TLR2, TLR3 was also shown to recognize cells undergoing necrosis during acute inflammatory events, independently of viral infection [39]. Indeed, as details of the mechanisms of endogenous TLR ligand recognition emerge, it becomes clear that there are significant differences between PAMP and DAMP activation of TLRs. We discuss these differences in detail in the following sections of this review. In addition, the phenotype of mice with targeted deletions in a number of endogenous TLR activators confirms that removal of endogenous danger signals correlates with the effects of addition of exogenous DAMPs. Together these data indicate that DAMP activity is not reliant on the presence of contaminating PAMPs. 

Recent data indicate that endogenous danger signals and microbial products can also cooperate in the induction of immune responses. Neither highly purified HSP preparations nor LPS alone at concentrations corresponding to those found in contaminated HSP preparations could induce pro-inflammatory cytokine production (reviewed in [40–42]). Further studies showed that HSP60 and Gp96 can tightly bind to LPS in a saturable manner and enhance its biological activity, as well as that of the TLR2 ligand Pam_3_Cys [43–45]. In the light of these results, the function of HSPs has been proposed to modulate early immune responses during infection by mediating a synergy between PAMPs and DAMPs. Similarly, HSP90 has also been implicated in the recognition of CpG DNA by TLR9 and the binding of HMGBs to nucleic acids is required for efficient recognition by TLR3, 7, and 9 [46–48].

## 3. Mechanisms of TLR Activation by DAMPs versus PAMPs

There is an increasing body of evidence that demonstrates how exogenous and endogenous activation of TLRs is mediated and this reveals that, whilst there is some overlap in molecular machinery, DAMPs possess distinct mechanisms of action to PAMPs. These similarities and differences emerge below where we explore the mechanisms of PAMP and DAMP recognition by TLRs and the subsequent TLR signalling and biological outcomes. 

### 3.1. Ligand Recognition by TLRs

#### 3.1.1. Exogenous Ligand Recognition

TLRs interact with a wide variety of ligands ranging from proteins and lipoproteins to nucleic acids and saccharides, all of which are different in size and chemical properties. The extracellular domains (ECDs) of TLRs contain leucine-rich repeat (LRR) motifs that are responsible for PAMP recognition [49]. The crystal structures of three TLR ECD-ligand complexes have been solved. One structure shows that TLR3 interacts with hydrophilic double-stranded RNA (dsRNA) via surface-exposed sites [50]. A second structure shows TLR1-TLR2 heterodimers bound to the hydrophobic Pam_3_CSK_4_ lipopeptide that fits in an internal hydrophobic pocket [51]. Finally, the structure of the TLR4-MD-2-LPS complex shows that TLR4 employs the co-receptor MD-2 to recognise LPS and that no direct contacts between the receptor and the ligand take place [52–54]. The latter structure also provided insights into the structure-activity relationship of the lipid A moiety of LPS. Not only the number of lipid chains [55, 56] but also the phosphate groups and their positioning in the lipid A are important factors affecting the immunological activity of LPS [53]. This suggests that even minor modifications to ligands may cause significant changes in the responses they generate. 

These three crystal structures highlight three diverse modes of exogenous ligand recognition by TLRs involving TLR homo- and heterodimerization as well as direct TLR-ligand interactions or the use of co-receptors and accessory molecules. A number of accessory molecules have been shown to assist microbial recognition by TLRs. For instance, LPS is extracted from the bacterial membrane by the LPS-binding protein (LBP) after which it is transferred to CD14. Subsequent transfer from CD14 to an additional accessory molecule MD-2 then allows TLR4-mediated LPS recognition [57]. Interestingly, in the absence of MD-2, the LPS-dependent TLR4 signalling can be reconstituted by the mite dust allergen Der p 2, which has structural and functional homology with MD-2 and mimics the activity of MD-2 by presenting LPS to TLR4 [58, 59]. HMGB1 can also mediate LPS transfer to CD14 to initiate a TLR4-mediated pro-inflammatory response [60]. In B cells, the TLR-like molecule radioprotective 105 (RP105) forms a complex with the MD-2 homolog MD-1 and is essential for regulating TLR2 and 4-dependent antibody production to the ligands lipoproteins and LPS. Conversely, in macrophages and DCs, RP105/MD-1 acts as a TLR4 decoy receptor that, by interacting directly with the TLR4 signalling complex, inhibits the ability of the receptor to bind microbial ligands [61, 62]. Other accessory molecules bind directly to TLR ligands. CD14 facilitates LPS transfer to TLR4/MD-2 and, accordingly, in the absence of CD14 rough LPS cannot initiate the TRIF/TRAM pathway and smooth LPS cannot be detected at all [63, 64]. CD14 binds also to triacylated lipopeptides facilitating their recognition by TLR2/TLR1 complexes [65] and can enhance dsRNA-mediated TLR3 activation [66]. CD36 is a sensor of diacylated lipopeptides recognised by TLR2/TLR6 [67]. NAD(P)H oxidase 4 (Nox4) modulates the production of LPS-induced reactive oxygen species (ROS) by interacting with the cytoplasmic TIR domain of TLR4 [68, 69]. TLRs also cooperate with other families of receptors to recognise microbial ligands. TLR2 was shown to collaborate with dectin-1 in zymosan recognition [70] or with the macrophage receptor with collagenous structure (MARCO) in addition to CD14 to respond to TDM, a cell wall glycolipid from *Mycobacterium tuberculosis* [71]. Collectively these data point to specific and complex mechanisms at the basis of PAMP recognition, highlighted by the requirement of a number of distinct co-receptors and accessory molecules for individual ligands.

#### 3.1.2. Endogenous Ligand Recognition by TLRs

No crystal structures of TLR-endogenous ligand complexes have been reported so far. Most of the proposed endogenous TLR activators have been shown to form complexes with TLRs *in vitro* by means of immunoprecipitation assays and functional cell-based assays or *in vivo*, taking advantage of mice deficient in TLRs or their adaptor proteins. Recently, FRET confocal microscopy and GFP fragment reconstitution have been proposed to study TLR interaction and measure distances between receptors in the range of molecular interactions [72]. This technique might be of great benefit in demonstrating and characterising endogenous ligand recognition by TLRs. 

There exists circumstantial evidence that DAMPs and PAMPs may occupy the same or neighbouring binding sites on TLRs. For instance, surfactant protein A was shown to downregulate peptidoglycan and zymosan induced NF*κ*B activation and TNF*α* secretion by binding to the extracellular domain of TLR2 in RAW 264.7 and alveolar macrophages [73, 74]. However, some DAMPs may utilize different binding sites; whilst the TLR4 mutations D299G and T399I prevent activation by LPS, these polymorphisms confer enhanced ability of TLR4 to respond to fibrinogen [75].

**Figure 2:**
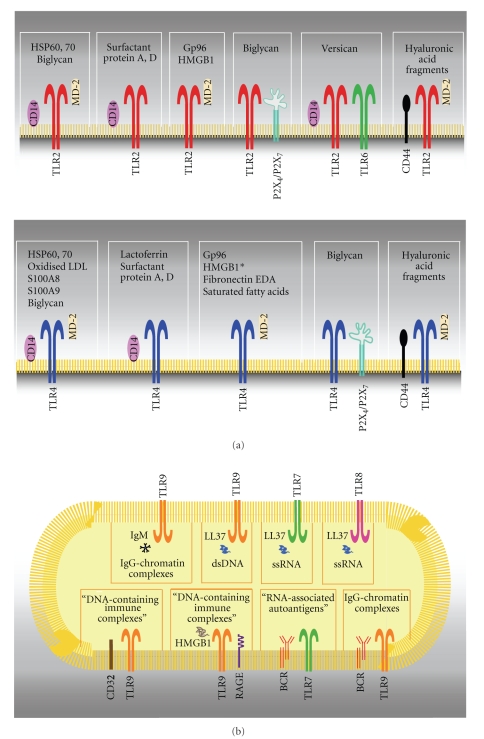
*Endogenous ligand recognition by TLRs*. The co-receptor(s) and accessory molecule(s) required by DAMPs for recognition by TLR(s) and subsequent cellular activation are shown. (a) TLRs localised on the plasma membrane; (b) TLRs resident in intracellular compartments. (*)HMGB1 may require MD-2 and CD14 for TLR4 activation (see [96]).

There is also evidence that DAMPs require different co-receptors and accessory molecules to PAMPs. Reviewing the proposed modes of endogenous ligand recognition leads to a rational classification of endogenous molecules based on the receptor, co-receptor(s), and accessory molecule(s) requirement for recognition by TLR(s) and subsequent cellular activation that is summarized in Figure 2. A first group of DAMPs requires both CD14 and MD-2. This includes both TLR2 and 4 agonists, such as HSP60, HSP70, and biglycan, as well as TLR4 activators such as oxidized LDL and S100 proteins [15, 23, 69, 76, 77]. A second group of DAMPs requires only CD14 as an accessory molecule and these are surfactant protein A and D and lactoferrin [78–80]. A third group comprises DAMPs that have been shown to involve only MD-2 in their recognition by TLRs. Among these, Gp96 and HMGB1 activate TLR2 and 4, whereas fibronectin EDA (FNEDA) and saturated fatty acids activate TLR4 [17, 20, 22, 81–86]. A fourth group includes DAMPs that use co-receptors or accessory molecules different from CD14 and MD-2. Biglycan was recently shown to induce the NLRP3/ASC inflammasome through activation of TLR2/4 and purinergic P2X_4_/P2X_7_ receptors [87]. Versican-induced responses require TLR2, TLR6, and CD14 [25]. HA dependent TLR4 activation involves CD44 in addition to MD-2 [88]. Autoantibodies against dsDNA and nucleosomes from SLE patients induce DC activation through TLR2 if bound to HMGB1 [89, 90]. Similarly, HMGB1 mediates the activation of plasmacytoid DCs and B cells through TLR9 by DNA-containing immune complexes through a mechanism involving the immunoglobulin superfamily member RAGE [46]. IgG2a-chromatin immune complexes require the synergistic engagement of IgM and TLR9 to activate B cells [31]. TLR7, 8, and TLR9 expressed by pDCs respond to self-RNA and -DNA respectively when coupled with the endogenous antimicrobial peptide LL37 [91, 92]. Furthermore, CD32 delivers DNA-containing immune complexes found in serum from SLE patients to intracellular lysosomes containing TLR9, leading to DC activation [89, 90]. Finally, B cells are activated by DNA- or RNA-associated autoantigens by combined B cell antigen receptor (BCR)/TLR9 or TLR7 engagement [93, 94]. This is a provisional list of endogenous activators and their accessory molecules that will certainly expand as we learn more about DAMP-TLR interactions. Collectively, these data indicate that several co-receptors and accessory molecules required for ligand recognition by TLRs are employed by both DAMPs and PAMPs. Further detailed investigation of how DAMPs are recognised by the cell is required to elucidate the precise structural organization of these receptor complexes. A signalling competent conformation of the receptor is required for TLRs to function; however it is not known whether the conformation induced by DAMPs is similar or distinct to that produced by microbial structures where sequential changes in receptor conformation occur upon ligand binding (reviewed in [95]).

### 3.2. TLR Signalling and Biological Outcomes

Ligand-induced receptor homo- or heterodimerization leads the cytoplasmic signalling domains of TLRs to dimerize. Despite diverse mechanisms of ligand interaction, PAMP-TLR complex crystal studies showed striking similarities in the organization of ligand-TLR dimer complexes that may apply to all TLRs. All three structures feature an “m”-shaped TLR dimeric architecture in which the C-terminal ends of the TLRs converge and, presumably, cause dimerization of the intracellular domains for signal initiation (reviewed in [97]). The resulting TIR-TIR complex initiates downstream signalling through recruitment of specific adaptor molecules. Five adaptors have been described so far: myeloid differentiation factor 88 (MyD88), MyD88-adaptor like (Mal), TIR domain-containing adaptor inducing IFN-beta (TRIF), TRIF-related adaptor molecule (TRAM), and sterile alpha and HEAT-Armadillo motifs (SARM) [98]. 

Depending on the adaptors recruited to the TLRs, two major intracellular signalling pathways can be activated by TLRs. The first, a MyD88-dependent pathway, is activated by all TLRs except TLR3. It involves the IL-1R-associated kinases (IRAK), IRAK-1 and IRAK-4, TNF receptor-associated factor 6 (TRAF-6), and mitogen-activated kinases (MAPK) and it culminates in the activation of the transcription factor NF*κ*B via the IkB kinase (IKK) complex. In turn, NF*κ*B mediates the transcription of pro-inflammatory cytokine genes. The second pathway, known as TRIF pathway, is independent of MyD88 and can be activated upon stimulation of TLR3 or 4. It leads to activation of the interferon-regulated factors (IRF) family of transcription factors via recruitment of TRIF and results in the synthesis of interferon (IFN).

TLR signalling pathways induced by endogenous molecules in different cell types are poorly investigated, but recent studies report usage of distinct adaptor molecules and induction of distinct signalling pathways downstream of TLRs when stimulated with exogenous or endogenous molecules. Activation of TLR4 by LPS can induce both TRIF and MyD88-dependent pathways. In contrast, we have shown that the endogenous TLR4 agonist tenascin-C signals via MyD88 [24]. Similarly, biglycan has been shown to signal through TLR2 and 4 in a MyD88-dependent manner [23]. 

TLR signalling results in the activation of transcription factors regulating the expression of specific genes whose products trigger various cellular responses. For example, NF*κ*B, AP-1, and IRF5 control the expression of genes encoding inflammatory cytokines, whereas IRF3 and IRF7 induce the expression of type I IFN and IFN-inducible genes. Thus a large number of proteins are synthesised that mediate inflammatory and immune responses and include inflammatory cytokines such as IL-1, IL-6, TNF*α*, IL-12, IFNs, chemokines, adhesion molecules, costimulatory molecules, growth factors, tissue-degrading enzymes such as metalloproteinases, and enzymes that generate inflammatory mediators such as cyclo-oxygenase 2 and inducible nitric oxide synthase (iNOS). 

Different microbial agents trigger multiple pathways in different cell types and induce the expression of distinct subset of genes [99–103]. A detailed comparative analysis of the biological outcomes induced by different endogenous versus exogenous TLR molecules has not been performed. However, some crucial differences between host responses to endogenous versus microbial agents are emerging. 

HMGB1 and LPS were shown to induce distinct patterns of gene expression in neutrophils. For instance, expression of monoamine oxidase B and the anti-apoptosis protein Bcl-xl was increased in neutrophils by HMGB1 but not by LPS. Furthermore, whilst the cytokine expression profile induced by HMGB1 versus LPS was similar, a slower induction of TNF*α* mRNA occurred upon LPS stimulation compared to HMGB1 [20, 104, 105]. HSP60 and LPS, in addition to synergistically enhancing IL-12p40 and IFN*γ* production in murine macrophages and in mHSP60-expressing COS1 cells, were shown to differentially activate APC function. Indeed, only HSP60 was able to stimulate the production of IFN*α* in peritoneal macrophages and bone marrow-derived DCs and IFN*α* release was not further increased by HSP60/LPS complexes [43]. Hyaluronan fragments generated following injury were reported to induce inflammatory responses distinct from LPS. A microarray analysis performed on the mouse alveolar macrophage cell line (MH-S) generated a list of genes that respond differently to hyaluronan and LPS. For instance, MMP13, TGF-*β*2, SOCS3, and other genes were induced exclusively by hyaluronan. There were also major differences in the cytokine profile induced. While some cytokines including TNF*α*, MCP-1, and RANTES were equally induced by both ligands, others, such as granulocyte macrophage-colony stimulating factor (GM-CSF), granulocyte colony-stimulating factor (G-CSF), and IL-1*α*, were significantly different [88]. We have shown that tenascin-C stimulated pro-inflammatory cytokine synthesis in primary human macrophages and synovial fibroblasts in a cell type specific manner, which was significantly different from LPS. Tenascin-C dose dependently induced TNF*α*, IL-6, and IL-8 production in human macrophages. However, it only induced IL-6 synthesis in synovial fibroblasts, whereas LPS induced both IL-6 and IL-8 [24]. Further investigation is required to fully define the differences in signalling pathways and gene expression induced by endogenous versus exogenous TLR activators. 

## 4. DAMPs and Disease

DAMPs are key danger signals that alert the organism to tissue damage and initiate the process of tissue repair. However, in addition to this physiological role in the response to tissue injury, there is evidence which indicates that endogenous TLR activators also contribute to the pathogenesis of many inflammatory and autoimmune diseases that are characterized by aberrant TLR activation.

### 4.1. High Levels of DAMPs Are Associated with Human Inflammatory Disease

**Figure 3:**
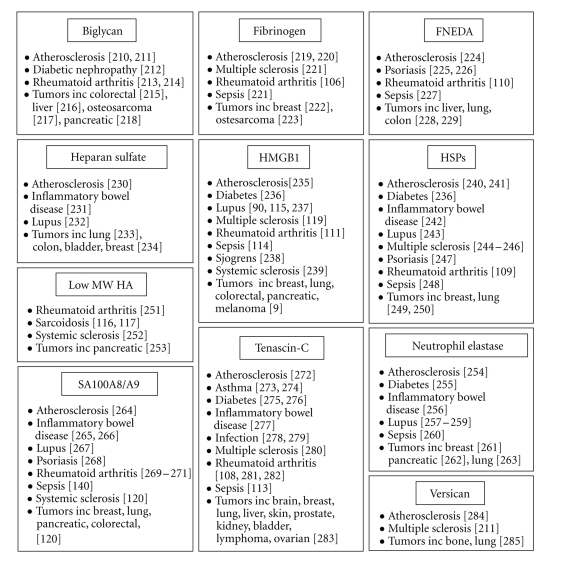
*DAMPs and human disease*. High levels of many DAMPs are associated with a wide variety of inflammatory and autoimmune diseases as well as with atherosclerosis and cancer.

The etiology of many inflammatory and autoimmune diseases is unclear; the initiating stimuli are often not well defined and the reasons why the mechanisms that ordinarily control the immune response fail are not known. However, it is clear that these diseases are characterized by an extremely destructive tissue environment. Accordingly, high levels of DAMPs occur locally and/or systemically in many of these conditions. For example, a wide range of endogenous TLR activators, including heat shock proteins, HMGB1, host DNA, fibrinogen, FNEDA, and tenascin-C, are observed in synovia of RA patients but not in synovia from normal joints or non-inflamed synovia from osteoarthritis (OA) patients [106–112]. High levels of HMGB1 and tenascin-C circulate in the serum of septic patients [113, 114], and high serum concentrations of DNA-containing immune complexes are associated with SLE [46], including nucleosome-HMGB1 complexes [90, 115]. In addition, elevated levels of low MW HA fragments are reported in the bronchial alveolar lavage fluid and serum of patients with inflammatory lung diseases [116–118]. In many cases levels of endogenous TLR activators are indicative of disease activity; elevated levels of extracellular HMGB1 localize specifically to active lesions of multiple sclerosis (MS) patients and correlate with active inflammation [119]. Furthermore, the S100 family of calcium binding proteins have long been reliable biomarkers of inflammation in a wide variety of diseases; for example, both MRP8 and MRP14 levels in the RA synovium and synovial fluid correlate with disease activity to a degree greater than levels of C-reactive protein (reviewed in [120]). Many endogenous TLR activators are also overexpressed in tumor cells. Figure 3 summarises some of the diseases with which endogenous TLR activators are associated.

### 4.2. Administration of Exogenous DAMPs Induces Inflammation In Vivo

Further support of a role for endogenous TLR activators in driving disease derives from *in vivo* studies using experimental models of inflammatory disease. Levels of many DAMPs are elevated during the pathogenesis of numerous diseases in rodent models. In addition, delivery of exogenous DAMPs promotes inflammation *in vivo* via activation of TLRs. Intra-articular injection of the TLR4 activators FNEDA or tenascin-C induces joint inflammation in wild type but not in TLR4 null mice [24, 86]. Systemic injection of HS causes lethal sepsis, similar to that induced by LPS or zymosan, in wild type but not in TLR4 null mice [121]. Furthermore, DNA released from necrotic hepatocytes stimulates cytokine synthesis via activation of TLR9 during murine acetaminophen-induced liver injury [122]. These and other studies are summarized in Table 1. In addition, many DAMPs can act as adjuvants; this has recently been comprehensively reviewed by Kono and Rock [6]. For example, purified genomic dsDNA boosted both antibody and CD8+ T cell responses in mice when injected with antigen [123]. Likewise lactoferrin, defensins, low MW HA, and HMGB1 all exhibit adjuvant properties *in vivo* [124–127]. Together these data show that many endogenous TLR activators exhibit pro-inflammatory properties *in vivo*.

### 4.3. Inhibition of DAMP Action In Vivo Ameliorates Disease

Whilst many DAMPs can induce TLR dependent inflammation *in vivo*, this does not necessarily demonstrate that these molecules are important in the progression of disease. This evidence has come from mice that do not express specific endogenous TLR activators (Table 2) and studies showing that inhibition of DAMP function can ameliorate disease *in vivo* (Table 3) and we review these data below.

#### 4.3.1. Targeted Deletion of DAMPs Protects from Inflammatory Disease

Biglycan null mice have a considerable survival benefit in LPS-induced sepsis due to reduced TLR2 and 4 dependent cytokine synthesis, cellular infiltration into tissues [23], and lower levels of active caspase-1 and mature IL-1*β* in the kidney, lung, and circulation [87]. We have shown that tenascin-C null mice are protected from persistent joint inflammation and tissue destruction during antigen-induced arthritis [24]. In addition, mice lacking MRP8/MRP14 complexes are protected from endotoxin-induced lethal shock and *E. coli*-induced abdominal sepsis [140, 143] and exhibit reduced lesion volume, brain swelling, and inflammatory cell infiltration during cerebral ischemia [142]. Furthermore, consistent with their enhanced expression during myocardial infarction, mice that lack MRP-8/14 complexes exhibited reduced inflammatory cell infiltration upon experimental arterial injury and attenuated atherosclerotic lesions and macrophage accumulation in plaques compared with mice deficient in apolipoprotein E alone [141].

#### 4.3.2. DAMP Antagonists Ameliorate Disease

The fact that blockade of DAMP function ameliorates disease *in vivo* further supports a role for endogenous TLR activators in inflammatory disease. The best example of how this can be achieved is with HMGB1 (reviewed in [170–174]), although manipulation of other DAMPs including HA, neutrophil elastase (NE), and versican can all protect against disease (Table 3). DAMPs comprise an enormously diverse subset of molecules. As such there exists a number of different mechanisms to prevent their inflammatory action, some of which are described below.

**(i) Blockade of TLR Activation**

One strategy that has proved effective is to manipulate the function of individual DAMPs by preventing TLR activation at the cell surface. Administration of polyclonal anti-HMGB1 antibodies or the DNA-binding A box of HMGB1, a competitive inhibitor of the pro-inflammatory B box, can reverse the lethality of established sepsis [114, 153, 154] and ameliorate collagen-induced arthritis in rodents (reviewed in [161]). However, whilst some reports demonstrate that monoclonal anti-HMGB1 antibodies are efficacious preventing organ damage in experimental models of sepsis [155], others suggest that monoclonal antibodies are not effective in suppressing arthritic disease *in vivo* [175]. This may be due to the multivalent nature of the mode of action of HMGB1. One alternative approach may be to use synthetic, bent oligonucleotides that have a high affinity for HMGB1, and suppress HMGB1-induced proliferation and migration of smooth muscle cells *in vitro* [176]. Another approach may be the use of an engineered mutant fragment, HMGB1 Mut (102–105) carrying two glycine substitutions, that decreased TNF*α*  release induced by the full-length HMGB1 protein in human monocyte cultures [177]. In addition, the N-terminal domain of thrombomodulin, an endothelial anticoagulant cofactor, exerts anti-inflammatory effects in a model of lethal endotoxemia partly by binding to and sequestering HMGB1 [178].

**(ii) Prevention of DAMP Accumulation**

DAMPs can be generated by release from necrotic cells, secretion from activated cells, cleavage of larger molecules, or specific upregulation upon tissue injury. Manipulation of tissue levels of DAMPs may provide another window of therapeutic opportunity. Indeed, ethyl pyruvate, stearoyl lysophosphatidylcholine, and nicotine have been shown to be efficacious in ameliorating experimental sepsis by preventing HMGB1 release during experimental sepsis [156–158]. However, the mechanism by which they do so is unclear and these compounds are likely also to affect numerous other cell processes. HMGB1 is released from cells by two distinct mechanisms: it is liberated from cells undergoing necrosis [179], or it is hyperacetylated and then actively secreted from stimulated cells. This non-classical secretion pathway is distinct from the passage through the ER and Golgi taken by signal tagged proteins, instead requiring the microtubule cytoskeleton [180]. Other DAMPs including the S100 proteins are also secreted in the same way [181] and targeting this pathway therefore may potentially offer a means to modulate the release of intracellular DAMPs.

One class of DAMPs comprises ECM fragments generated by release from intact matrices. Inhibition of this process has been demonstrated; for example, release of immune-stimulatory HS fragments from the ECM *in vivo* can be mediated by the proteolytic action of elastase [182]. Injection of elastase into the peritoneal cavity of mice caused the release of HS and induced sepsis, nearly as effectively as direct injection of HS or LPS [121]. Thus therapeutic measures aimed at blocking elastase could reduce the production of endogenous TLR4 activators. Indeed, pre-treatment with NE inhibitor before induction of hepatic ischemia-reperfusion injury ameliorated liver damage [168]. HS fragments are also generated upon ECM oxidation by reactive oxygen species (ROS). Extracellular superoxide dismutase (EC-SOD) is an antioxidant enzyme that protects the lung from oxidant-mediated inflammation. One way in which it does this is to protect HS from oxidative fragmentation; bronchoalveolar lavage fluid from EC-SOD knockout mice after asbestos exposure showed increased HS shedding from the lung parenchyma [183]. An alternative strategy may be to alter the balance of immune-silent intact ECM molecules versus immune-stimulatory fragments either directly or indirectly. Specific over expression of high MW HA in the lung has been achieved using transgenic mice that constitutively express HA synthase. These mice showed that improved survival and decreased apoptosis during bleomycin induce lung inflammation [145].

Finally, for DAMPs whose expression is specifically upregulated during inflammation it may be possible to manipulate this induction of expression. Indeed, knockdown of versican expression in Lewis lung carcinoma cell lines (LLC) ablated their tumorigenic capability, promoting mouse survival and reduced metastasis, whilst overexpression of versican in LLC lines with low innate metastatic potential increased lung metastasis [25].

Together these data indicate that endogenous TLR activators significantly contribute to driving inflammatory disease *in vivo* and suggest that targeting this method of TLR activation may potentially be of therapeutic value in combating disease.

## 5. Conclusions and Perspectives: Targeting DAMPs in the Clinic?

Current strategies in clinical development for TLR blockade include (1) global blockade of individual TLR function using neutralizing antibodies, soluble TLR extracellular domains (ECDs), natural antagonists, and small molecule inhibitors, (2) inhibition of signalling pathways-activated downstream of TLR stimulation using small molecules to target MyD88/TRAF/IRAK complex formation, MAPK, or IKK activity, or (3) using PAMP antagonists such as LPS inhibitors. Some of these compounds have reached phase II clinical trials and the results are currently awaited, whilst others, particularly those targeting common signalling pathways such as MAPK, have proved to be of limited efficacy (reviewed in [11]). 

Suppressing DAMP activation of TLRs offers a host of new potential targets for treating inflammatory diseases that may be viable alternatives to current approaches. Evidence that blockade of these mediators can ameliorate disease in human studies is beginning to emerge. Hyaluronate improves pain and prostaglandin E (PGE) levels in patients with RA [184], transfer of HSP-specific regulatory T cells inhibits inflammation in animal models of arthritis and exhibited promising results in preliminary clinical trials [185], HMGB1 antibodies prevent the activation of cells by serum from SLE patients [46], and the neutrophil elastase inhibitor sivelestat improves the mortality rate of patients with sepsis [186, 187]. By carefully choosing a target unique to the response to tissue damage, and not to pathogen mediated activation of the immune response, this strategy may have the additional advantage of leaving the host response to infection intact. Given the evidence that supports the idea that distinct molecular machinery is required for DAMP activation of TLRs, another strategy would be to block co-receptors or accessory molecules essential for DAMP activation. In addition a comparative analysis of adaptors, kinases, and transcription factors involved in signalling activated by DAMPs versus PAMPs may highlight key differences that, if selectively targeted, could lead to specific therapies engineered to silence danger signals without compromising the host immune defence. 

We have highlighted here the possible levels of intervention in DAMP activation of TLR-mediated inflammation, namely, manipulating DAMP activation of TLRs or controlling tissue level of DAMPs. Whilst these strategies are efficacious in preventing experimental disease, there is also evidence that preconditioning with DAMPs can have the same effect. Administration of small doses of HMGB1 one hour prior to induction of hepatic reperfusion injury protected from liver damage and reduced serum TNF*α* and IL-6 levels via inhibition of TLR4 signalling [188, 189] and lactoferrin can protect from lethal *E.coli* injection [190]. 

**Figure 4:**
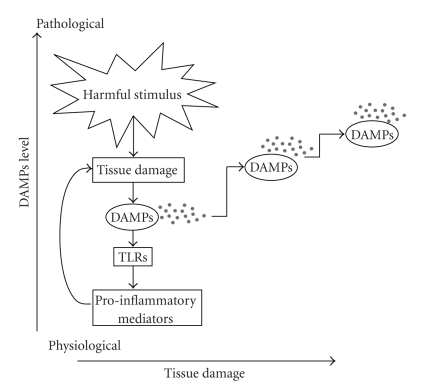
*The “damage chain reaction.”* Harmful stimuli, including pathogens, injury, heat, autoantigens, tumor, and necrotic cells, cause tissue damage. Endogenous danger signals are generated and induce a pro-inflammatory cascade by activating TLRs. In turn, pro-inflammatory mediators are upregulated and trigger further tissue damage leading to increasing DAMPs levels. Thus a vicious cycle is sustained and may result in chronic inflammation and autoimmunity.

It is apparent that low tissue levels of DAMPs are beneficial during tissue repair to induce a resolvable, physiological immune response. In contrast, high levels of DAMPs are generated during chronic inflammation. We propose a situation where a damage chain reaction occurs: increasing levels of pro-inflammatory DAMPs create more tissue damage which significantly amplifies the tissue levels of DAMPs which go on to create yet more tissue damage ad infinitum. These tissue levels of DAMPs become harmful and mediate a non-resolving perpetual inflammatory state (Figure 4). Thus targeting DAMP-mediated activation of TLRs may block this chronic inflammatory loop, although it will be important to assess whether total blockade of DAMP function will compromise tissue repair to any deleterious extent. 

In addition, in the destructive milieu that occurs during inflammatory disease there are likely to be high levels of many DAMPs. Working out which are keys to disease pathogenesis may not be a trivial matter, and combinations of inhibitors may be needed to successfully dampen down endogenously driven inflammation. Alternatively, hierarchies may exist amongst DAMPs such as those that exist for inflammatory cytokines, for example, where TNF*α* induces a cascade of cytokine synthesis. Indeed, low MW HA induces *β*-defensin2 via TLR2 and 4 activation in murine keratinocytes *ex vivo* and *in vivo* [191]. These DAMPs may be key targets to prevent the induction of an autocrine loop of inflammation. Unravelling potential hierarchies amongst DAMPs represents a major challenge for future investigations. These may be aided by approaches such as microarray and deep sequencing technologies, as well as proteomewide screening, to enable the comparison of the global effects of different DAMPs on inflammatory gene expression. Likewise, examining the stimuli that induce DAMP expression or release upon tissue injury will be important in establishing a “chain of command” of DAMP action. In parallel, the development of validated reagents and tools which serve to ablate the expression or function of individual DAMPS will yield key information about redundancy and co-dependency.

The threshold of DAMP(s) required to induce disease may vary upon the duration and degree of host tissue damage. However, current knowledge about the kinetics of expression or release of DAMPs and their turnover during disease progression is limited. Indeed, on one hand, validated commercial assays for measuring various endogenous danger signals are often unavailable or prohibitively expensive. On the other hand, access to patient specimens and pathological data is in many cases restricted. Thus, the correlation between degree of tissue damage and levels of DAMP(s) is either unknown or limited to small sample size, often representative of end stage of disease. The threshold of DAMPs required to trigger chronic inflammation may also depend on a variety of host genetic factors, including single-nucleotide polymorphisms (SNPs), which can affect how humans respond to injury and develop disease. Examining the role of DAMPs within the context of different genetic backgrounds will also be key to dissecting out their role in inflammatory disease. The use of larger patient sample sizes including diverse genetic populations and a befitting proportion of male and females will be vital. In addition, the development of mouse strains with much greater DNA diversity than strains traditionally employed [192] may provide mice with combinations of different traits that more closely reflect the genomic variations of humans in preclinical studies. We expect that the next few years will provide a much more concrete picture of how DAMPs link tissue damage to chronic inflammation as an increasing number of tools become available. 

Finally, a normal wound healing response does not typically lead to chronic inflammation. This is, in part, because a number of mechanisms exist to negatively regulate TLR activation. These include the release of soluble decoy TLR receptors, intracellular inhibitory molecules such as IRAK-M, SOCS1, Tam family kinases, and transmembrane regulators such as SIGIRR (reviewed in [8, 193]). Viruses have also evolved mechanisms to target adapters in TLR signalling: A46R from vaccinia virus, which sequesters MyD88, Mal, Trif, and Tram [194], and NS3/4A from hepatitis C virus, which degrades Trif [195]. In addition, recently, microRNA, miR-147, whose expression is induced upon stimulation of multiple TLRs, was shown to attenuate TLR stimulation-induced-inflammatory response in macrophages [196].

However, these pathways do not appear to discriminate between distinct methods of TLR stimulation and act on DAMP- and PAMP-mediated activation alike. Chen et al. recently identified one way in which specific activation of TLRs by DAMPs, but not PAMPs, may be inhibited (reviewed in [197]). CD24, or heat stable antigen, is a GPI anchored protein that binds to DAMPs such as HMGB1, hsp70, and hsp90 in order to suppress their activation of inflammatory signalling pathways. CD24 null mice exhibit increased susceptibility to DAMP-, but not PAMP, induced inflammation. This is mediated at least in part through CD24 association with Siglec-10/G causing activation of associated phosphatases which are proposed to repress DAMP-initiated signalling. Dysfunction of this pathway might contribute to the etiology of autoimmune diseases and likewise may offer a means to selectively inhibit DAMP activity [198]. In addition, sTLR2 can blunt immune responses without preventing microbial recognition: mice injected with Gram positive bacteria together with sTLR2 exhibited reduced inflammatory cytokine levels and cell migration but this did not compromise their ability to clear live Gram-positive bacteria-induced infection [199]. As such enhancing naturally suppressive mechanisms may also be a viable strategy for reducing inflammation.

Thus DAMPs appear to be a double-edged sword. While being vital for tissue repair, they also play a role in the pathogenesis of many inflammatory and autoimmune diseases that feature aberrant TLR activation. In these diseases harmful stimuli cause tissue damage; in an attempt of tissue healing, inflammatory responses are initiated and generate DAMPs that induce an autocrine loop of inflammation. Understanding why the natural mechanisms that keep DAMP-mediated inflammation in check fail in disease, as well as dissecting out which mechanisms of TLR activation and signalling are unique to DAMPs, may enlighten our approach to engineering targeted and efficacious therapies designed to dampen inflammation.

## Figures and Tables

**Table 1:** *DAMPs induce disease in vivo.* Administration of DAMPs to rodents either intra-articularly (i.a.), intracerebroventricularly (i.c.), intraperitoneally (i.p.), intratracheally (i.t.), or intravenously (i.v.) can provoke pathological inflammation *in vivo*.

Pathology	DAMP	Effect	Refs
Atherosclerosis	Apo CIII-rich VLDL (i.v.)	Stimulated TLR2 dependent monocyte activation and adhesion	[128]

Brain injury	HMGB1 (i.c.)	Increased cytokines, taste aversion, fever, mechanical allodynia, promotes severity of infarction	[129–131]

Gut inflammation	HMGB1 (B box) (i.p.)	Increased ileal mucosal permeability and bacterial translocation to mesenteric lymph nodes	[132]

Joint disease	FNEDA (i.a.)	Induced TLR4 dependent transient ankle swelling, cytokine synthesis, synovial inflammation	[86]
	HMGB1 (i.a.)	Induced synovial inflammation, some pannus formation	[133]
	Tenascin-C (i.a.)	Induced TLR4 dependent joint inflammation and tissue erosion	[24]

Liver injury	DNA	During acetaminophen induced cell death induced TLR9 dependent tissue injury	[134]
	HMGB-1 (i.p.)	Aggravated ischemic reperfusion injury	[135]

Lung injury	HMGB-1 (i.t.)	Stimulated acute inflammatory injury, neutrophil accumulation, edema, cytokine production	[136–138]

Sepsis	HS (i.p.)	Induced TLR4 dependent lethality	[121]
	HMGB1 (i.p.)	Induced partially TLR4 dependent lethality	[114]

**Table 2:** *Targeted deletion of DAMPs protects from experimental disease*. Mice which do not express certain DAMPs exhibit reduced symptoms of inflammatory disease or tumor metastasis *in vivo*.

DAMP	Disease Model	Effect of deletion	Refs
Biglycan	Renal inflammation (unilateral ureteral obstruction)	Reduced kidney damage	[87]
	Sepsis (LPS or zymosan)	Protected from lethality	[23, 87]

FNEDA	Atherosclerosis (Apo E deficient, high fat diet)	Reduced lesion size, number and macrophage infiltration	[139]

MRP8/MRP14	Abdominal sepsis (*E. coli*)	Reduced bacterial dissemination, systemic inflammation, liver damage	[140]
	Arterial injury (femoral wire insertion)	Reduced inflammatory cell infiltration and neotintimal formation	[141]
	Atherosclerosis (Apo E deficient, high fat diet)	Attenuated atherosclerotic lesions and macrophage accumulation in plaques	[141]
	Cerebral ischemia (cerebral artery occlusion)	Reduced lesion volume, brain swelling and inflammatory cell infiltration	[142]
	Lethal sepsis (LPS )	Protects from lethality	[143]
	Vasculitis (cytokine induced)	Reduced neutrophil accumulation and lesion severity	[141]

Tenascin-C	Destructive joint inflammation (antigen induced arthritis)	Protected from joint erosion and tissue destruction	[24]
	Tumorigenesis (cross with MMTV/PyV mice)	Induced smaller tumor nests	[144]

**Table 3:** *Manipulation of DAMP function ameliorates experimental disease*. Therapeutic blockade of DAMP function, for example, using monoclonal or polyclonal antibodies (mAb, pAb) or specific inhibitors or silencing DAMP expression by siRNA can reduce disease progression *in vivo*.

DAMP	Disease Model	Mechanism of blockade	Refs
HA	Lung inflammation (bleomycin)	Over expression of HA synthase improved survival and decreased apoptosis	[145]
	Sepsis (LPS)	High MW HA reduced lung neutrophil infiltration and cytokine synthesis	[146]

HMGB1	Brain injury (transient ischemia)	mAb reduced infarct size and severity, locomotor function, cytokine synthesis	[130]
	Colitis (DSS, TNBS)	pAb, ethyl pyruvate ameliorated disease, reduced cytokine synthesis, associated tumors	[147, 148]
	Lung inflammation (LPS)	pAb decreased neutrophils, lung edema	[136, 138]
	Lung injury (ventilator)	Ab improved oxygenation, neutrophil influx, cytokine synthesis	[149]
	Hepatic ischemia reperfusion injury	pAb decreased liver damage	[135]
	Acute pancreatitis (duodenal loop closure)	pAb improved pancreas and lung damage, aggravated bacterial translocation to pancreas	[150]
	Hemorrhagic shock (blood withdrawal)	pAb improved survival, ameliorated ileal permeability, decreased serum cytokines	[151]
	Hemorrhagic lung injury (cyclo-phosphamide)	pAb reduced pulmonary edema, neutrophil accumulation, lung permeability	[152]
	Sepsis (LPS, *E. Coli*, cecal ligation and puncture)	pAb, mAb, DNA binding box A protected from/reversed lethality, rescues taste aversion	[114, 129, 153–155]
		Ethyl pyruvate, stearoyl lysophosphatidylcholine, nicotine (inhibit secretion) protected from lethality	[156–158]
		Neural ant-inflammatory peptides vasoactive intestinal peptide and urocortin rescue lethality	[159]
		Cisplatin (nuclear sequestration of HMGB1) protected from lethality	[160]
	Rheumatoid arthritis (Collagen)	pAb, DNA binding box A reduced severity of established joint disease	[161]

HSP90	Rheumatoid arthritis (Collagen, adjuvant)	SNX-7081 (inhibitor) ameliorated disease, joints returned to normal	[162]
	Tumorigenesis (nude mice)	BX-2819 (inhibitor) inhibited tumor growth	[163]

Neutrophil elastase	Acute lung injury (LPS induced)	Sivelestat or L-658,758 (inhibitors) reduced HMGB levels and lung damage	[164, 165]
	Colitis (dextran)	ONO-5046 (inhibitor) reduced disease severity	[166]
	Rheumatoid arthritis (Collagen)	ONO-5046 (inhibitor) reduced incidence and severity of disease, ablated cartilage destruction	[167]
	Hepatic ischemia reperfusion injury	GW311616A (inhibitor) ameliorated liver damage, decreased neutrophil infiltration	[168]

Serum amyloid A	Tumorigenesis (LLC inoculation)	pAb reduced lung metastasis	[169]

Versican	Tumorigenesis (LLC inoculation)	siRNA reduced lung metastasis	[25]
